# Paclobutrazol treatment as a potential strategy for higher seed and oil yield in field-grown *camelina sativa *L. Crantz

**DOI:** 10.1186/1756-0500-5-137

**Published:** 2012-03-13

**Authors:** Sumit Kumar, Sreenivas Ghatty, Jella Satyanarayana, Anirban Guha, BSK Chaitanya, Attipalli R Reddy

**Affiliations:** 1 Department of Plant Sciences, University of Hyderabad, Hyderabad 500046, India; 2 Tree Oils India Limited (TOIL), Zaheerabad 502226, India; 3 Department of Environment, A.N.G.R. Agricultural University, Hyderabad 500030, India

**Keywords:** Biofuel *Camelina sativa *Growth Paclobutrazol Physiology Seed yield

## Abstract

**Background:**

*Camelina (Camelina sativa *L. Crantz) is a non-food oilseed crop which holds promise as an alternative biofuel energy resource. Its ability to grow in a variety of climatic and soil conditions and minimal requirements of agronomical inputs than other oilseed crops makes it economically viable for advanced biofuel production. We designed a study to investigate the effect of paclobutrazol [2RS, 3RS)-1-(4-Chlorophenyl)-4,4-dimethyl-2-(1H-1,2,4-triazol-1-yl)pentan-3-ol] (PBZ), a popular plant growth regulator, on the seed and oil yield of *Camelina sativa *(cv. Celine).

**Results:**

A field-based micro-trial setup was established in a randomized block design and the study was performed twice within a span of five months (October 2010 to February 2011) and five different PBZ treatments (Control: T_0_; 25 mg l^-1^: T_1_; 50 mg l^-1^: T_2_; 75 mg l^-1^: T_3_; 100 mg l^-1^: T_4_; 125 mg l^-1^: T_5_) were applied (soil application) at the time of initiation of flowering. PBZ at 100 mg l^-1 ^concentration (T_4_) resulted in highest seed and oil yield by 80% and 15%, respectively. The seed yield increment was mainly due to enhanced number of siliques per plant when compared to control. The PBZ - treated plants displayed better photosynthetic leaf gas exchange characteristics, higher chlorophyll contents and possessed dark green leaves which were photosynthetically active for a longer period and facilitated higher photoassimilation.

**Conclusion:**

We report for the first time that application of optimized PBZ dose can be a potential strategy to achieve higher seed and oil yield from *Camelina sativa *that holds great promise as a biofuel crop in future.

## Background

Global energy use and the carbon emissions during energy production are the major causes of climate change. A major challenge of the 21^st ^century is shifting our energy supply from carbon positive fossil-based petroleum to carbon neutral plant/algae-based fuels without affecting global food supplies. Rising fossil fuel prices, increasing energy demand and future projected shortages of transportation fuels have piqued greater interest in the production of green diesel from non-edible plant oils. Alternative diesel fuels have been primarily produced from soybean, oil palm and canola. However, due to economic non-viability as well as the recent crisis of food *vs*. fuel, the credibility of usage of edible oils as fuel has become the topic of controversy and debate. Hence exploration of less expensive alternative fuels has been necessitated. Further, in recent years, there has been resurgence of interest in exploiting non-edible oilseed crops with low inputs of water, fertilizers, pesticides and energy as well as for crops well suited to marginal soils for optimum yields. Non-edible oils have become the premier raw materials for biodiesel production. Many tree-borne oilseed species including *Jatropha curcas, Pongamia pinnata, Madhuca indica, Calophyllum inophyllum, Simarouba glauca *and *Azadirachta indica *are being investigated for the production of biodiesel. In addition, search is on for low cost and short rotation non-edible oilseed crops for improved and sustainable biodiesel production.

*Camelina sativa *(L.) Crantz. a member of Brassicaceae family, also known as gold of pleasure or false flax, is a winter annual, which can be grown in a wide range of soil conditions as spring or summer annual in temperate countries or as annual winter crop under tropical climate [1]. Biodiesel from *Camelina *oil has been successfully flight tested by Japan airlines (JAL) and KLH Royal Dutch airlines [2]. *Camelina *cultivation is much easier on marginal soils with low rainfall and fewer inputs compared to most other oilseed crops. However, the agronomic and crop production practices for *Camelina *have not been elucidated. Hence, it is important to understand the growth, development and yield improvement aspects for sustainable *Camelina *cultivation. *Camelina *is just being domesticated and it is exhibiting potential yield and oil content which are comparable to many other agronomically acceptable biofuel crops. Most importantly, the oil content in *Camelina *seeds is reported to range from 38-44% by dry weight while the content of crude protein is in the range of 25-45% [3].

One of the potential approaches to improve agronomic productivity is to manipulate the physiology of plants by application of plant growth regulators as observed in some of the recent studies on *Jatropha curcas *[4,5]. Paclobutrazol [2RS, 3RS)-1-(4-Chlorophenyl)-4,4-dimethyl-2-(1H-1,2,4-triazol-1-yl)pentan-3-ol] (PBZ) is a synthetic plant growth regulator, which has been used to control vegetative growth and to enhance flowering and fruiting patterns for obtaining improved economic yields of several tree crops. PBZ is known to inhibit gibberellin biosynthesis and can cause several physiological changes in plants including increased photosynthetic pigments, improved nutrient uptake, senescence retardation and enhanced flowering and seed yields [6]. Because of several positive effects of PBZ on several tree crops as well as certain annuals, we proposed this study to investigate the effects of PBZ on growth, physiology, seed and oil yields in *Camelina*. This paper is the first report on the use of PBZ as a potential strategy to significantly increase the seed and oil yields of *Camelina *under tropical semi-arid climatic conditions.

## Results

### Effect of PBZ on photosynthetic leaf gas exchange

**Figure 1:**
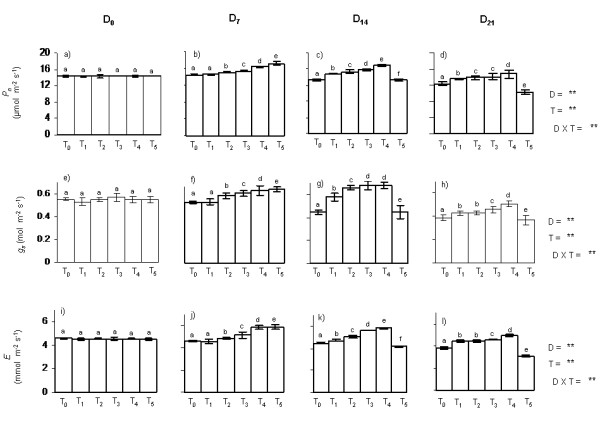
**Changes in P_n_, g_s _and E in *Camelina sativa *under five different soil treatments of Paclobutrazol**. Changes in (a-d) net photosynthetic rate (*P_n_*), (e-h) stomatal conductance to CO_2 _(*g_s_*) and (i-l) transpiration rate *E *in *Camelina sativa *under five different soil treatments of PBZ (Control: T_0_; 25 mg l^-1^: T_1_; 50 mg l^-1^: T_2_; 75 mg l^-1^: T_3_; 100 mg l^-1^: T_4_; 125 mg l^-1^: T_5_) as recorded on different time interval (day 0: D_0_; day 7: D_7_; day 14: D_14_; day 21: D_21_) of experimental period. Leaf gas exchange measurements were done on healthy leaves at the 3^rd ^to 4^th ^position from the top between 9:00-11:00 solar hours at a saturating PAR of 1500 μmol mol^-2 ^sec^-1^. Results are represented as mean of two trials ± SD of n = 6-15. Data were analyzed using two way analysis of variance (ANOVA) to test significant differences within days (D), among the treatments (T) as well as for their interactions (D × T) on measured traits (** significant at p < 0.01 level). Different letters indicate that the values are significantly different.

PBZ treatments induced apparent changes in leaf gas exchange characteristics of *Camelina *plants in both trials as represented in Figure 1. On D_0_, *P_n _*of the plants was recorded to be ~14.35 μmol m^-2 ^s^-1 ^(Figure 1a). With progression in time course and age of plants, an initial increase and thereafter a decreasing trend in *P_n _*was recorded in the control (T_0_) plants (Figure 1b, c, d). T_1 _and T_2 _caused a marginal, however significant (p < 0.05) increment in *P_n_*, as measured during three major stages of time course (D_7_, D_14 _and D_21_) when compared to corresponding control plants. On D_14 _and D_21_, *P_n _*values of T_1 _plants were 14.77 and 13.68 μmol m^-2 ^s^-1 ^respectively, whereas in case of T_2 _plants *P_n _*was found to be 15.38 and 13.88 μmol m^-2 ^s^-1 ^on D_14 _and D_21_, respectively (Figure 1b, c, d). In case of T_3 _and T_4_, plants displayed significantly higher *P_n _*in all growth stages of study when compared to other treatments (T_0_, T_1 _and T_2_) and the highest *P_n _*of 16.96 μmol m^-2 ^s^-1 ^was recorded in T_4 _plants followed by T_3 _(15.94 μmol m^-2 ^s^-1^) on D_14_. However, like T_0_, T_1 _and T_2_, the *P_n _*values exhibited same trend of down regulation in T_3 _and T_4 _plants with further progression in plant age and time course. The T_5 _plants showed maximum *P_n _*(17.2 μmol m^-2 ^s^-1^) on D_7 _(Figure 1b), and thereafter exhibited apparent reduction in *P_n _*on progressive days when compared to the plants of corresponding growth stages in other treatments (T_0_/T_1_/T_2_/T_3_/T_4_). On D_21_, the T_5 _plants showed the lowest *P_n _*of 10.3 μmol m^-2 ^s^-1 ^with a reduction of 28% when compared to the control (Figure 1d). The *P_n _*values showed statistically significant difference (p < 0.05) within days (D), among the treatments (T) and also the interaction D × T was significant.

On D_0_, *g_s _*of the control plants displayed mean rate of 0.55 mol m^-2 ^s^-1 ^(Figure 1e). In case of T_0_, between D_7 _and D_21_, *g_s _*ranged from 0.58 (D_7_) to 0.39 mol m^-2 ^s^-1 ^(D_21_) depicting a decreasing trend in *g_s _*with increase in plant age and progression of time course (Figure 1f, g, h). The T_1 _and T_2 _plants displayed enhanced *g_s _*on D_7 _(0.56 and 0.62 mol m^-2 ^s^-1^, respectively) and on D_14 _(0.58 and 0.66 mol m^-2 ^s^-1^, respectively). On D_21_, *g_s _*was significantly down regulated in both T_1 _and T_2 _plants when compared to earlier stages of growth (decreased to ~0.43 mol m^-2 ^s^-1^, p < 0.05) (Figure 1f, g, h). Compared to the other treatment, the T_4 _plants exhibited significantly (p < 0.05) higher *g_s _*in all the stages of gas exchange measurements and elucidated the maximum *g_s _*(~0.68 mol m^-2 ^s^-1^) on D_14 _(Figure 1g) and minimum *g_s _*of 0.51 mol m^-2 ^s^-1 ^on D_21 _(Figure 1h). The T_5 _plants initially elucidated significantly (p < 0.05) higher *g_s _*on D_7_; however with increase in plant age and progression in time course, T_5 _plants displayed substantial reduction in *g_s _*which decreased to 0.37 mol m^-2 ^s^-1 ^on D_21 _(Figure 1h). The *g_s _*values exhibited statistically significant differences (p < 0.05) within days (D), among the treatments (T) and the interaction D × T was also significant.

Similar to *g_s_*, PBZ application also enhanced the *E *of treated plants. On D_0_, the *E *values recorded were ~4.53 mmol m^-2 ^s^-1 ^(Figure 1i), thereafter the T_0 _plants maintained higher rates of *E *upto D_14 _(~4.57 mmol m^-2 ^s^-1^) and the rate decreased to 3.82 mmol m^-2 ^s^-1 ^on D_21 _(Figure 1l). In case of T_1 _and T_2_, plants showed marginal, however significant (p < 0.05) increment in *E *on D_7 _and D_14 _when compared to the controls of corresponding growth stages. On D_21_, *E *decreased to 4.43 and 4.44 mmol m^-2 ^s^-1 ^in T_1 _and T_2 _plants, respectively (Figure 1l). Compared to T_0_, T_1 _and T_2_, T_3 _and more specifically T_4 _plants manifested significantly (p < 0.05) higher *E *during D_7_, D_14 _and D_21 _stages of time course (Figure 1j, k, l). T_4 _plants showed maximum *E *(5.88 mmol m^-2 ^s^-1^) on D_14 _and minimum (4.96 mmol m^-2 ^s^-1^) on D_21_. The T_5 _plants displayed maximum E of 5.88 mmol m^-2 ^s^-1 ^on D_7_, however with increase in age, T_5 _plants exhibited substantial decrease (p < 0.05) in *E *and finally reached to 3.08 mmol m^-2 ^s^-1 ^of *E *on D_21 _(Figure 1l). In our study, the *E *values significantly differed (p < 0.05) within days (D), among the treatments (T) and also the interaction of D × T was found to be significant.

### Effect of PBZ on chlorophyll and carotenoid contents

All concentrations of PBZ significantly increased chlorophyll contents compared with the controls for both the trials (Table 1). In contrast to the PBZ treated plants, T_0 _did not show any significant changes in chlorophyll a contents at different time intervals. On D_14_, chlorophyll a contents in T_1 _and T_2 _plants showed maximum increment and was recorded to be ~30% higher with respect to the control. The T_3 _and T_4 _plants exhibited maximum changes in chlorophyll a contents and showed statistically significant increase of 50.9% (on D_21_) and 62.7% (on D_21_), respectively when compared to controls (T_0_). We observed significant (p < 0.05) difference in chlorophyll a contents of PBZ treated plants within the days (D), among the treatments (T) and between the interaction of D × T (Table 1). A similar effect of PBZ treatments was observed on chlorophyll b content. In case of T_1 _and T_2_, plants showed a marginal, however significant increase of ~6.3% and 12.9% in chlorophyll b contents on D_14_, respectively when compared to the control. T_3 _and T_4 _exhibited highest increment of 18.8% and 21.9% in chlorophyll b content on D_14, 21 _and D_14_, respectively compared with the control. Total chlorophyll was found to be increased significantly due to PBZ treatments and showed highest increment of 52.9% in T_4 _plants (on D_21_), followed by T_3 _(43.3% on D_21_), T_5 _(36.4% on D_14_), T_2 _(25.4% on D_14_) and T_1 _(24.6% on D_14_) when compared to the control (T_0_) plants. A significant difference (p < 0.05) was detected in the total chlorophyll content of the PBZ treated plants within the days (D), among the treatments (T) and between D × T. Plants treated with PBZ showed increased chlorophyll a/b ratio with progression in plant age and time course. Highest chlorophyll a/b was recorded in T_4 _plants on D_21 _followed by T_5 _(on D_21_), T_3 _(on D_14_), T_2 _(on D_21_) and T_1 _(on D_21_) plants. Both in PBZ treated as well as untreated control (T_0_) plants, total carotenoid content was found to increase with plant age and time course. Total carotenoid content significantly differed (p < 0.05) in the plants within the days (D), however the difference among the treatments (T) and D × T interaction were not found to be statistically significant (Table 1).

**Table 1:** Treatment effects of paclobutrazol on chlorophylls and carotenoid contents in the leaves of *Camelina sativa*

Treatment	Day of treatment	Chlorophyll a (mg g^-1 ^fw)	Chlorophyll b (mg g^-1 ^fw)	Total Chlorophyll (mg g^-1 ^fw)	Chlorophyll a/b	Total carotenoids (mg g^-1 ^fw)
**T_0_**	0	1.02 ± 0.02	0.32 ± 0.04	1.34 ± 0.06	3.22 ± 0.41	0.52 ± 0.04
	7	1.04 ± 0.01	0.32 ± 0.02	1.35 ± 0.02	3.35 ± 0.06	0.53 ± 0.05
	14	1.03 ± 0.20	0.32 ± 0.02	1.33 ± 0.20	3.21 ± 0.51	0.65 ± 0.03
	21	1.02 ± 0.10	0.31 ± 0.01	1.33 ± 0.12	3.28 ± 0.17	0.70 ± 0.06
**T_1_**	0	1.02 ± 0.01	0.32 ± 0.01	1.34 ± 0.01	3.18 ± 0.08	0.53 ± 0.05
	7	1.02 ± 0.04	0.32 ± 0.01	1.35 ± 0.05	3.19 ± 0.21	0.54 ± 0.04
	14	1.33 ± 0.10	0.34 ± 0.03	1.67 ± 0.08	3.93 ± 0.53	0.64 ± 0.04
	21	1.30 ± 0.05	0.33 ± 0.03	1.64 ± 0.06	3.93 ± 0.08	0.68 ± 0.03
**T_2_**	0	1.01 ± 0.03	0.31 ± 0.06	1.33 ± 0.09	3.23 ± 0.61	0.53 ± 0.01
	7	1.23 ± 0.05	0.33 ± 0.01	1.56 ± 0.05	3.73 ± 0.28	0.54 ± 0.03
	14	1.33 ± 0.10	0.35 ± 0.09	1.68 ± 0.08	3.82 ± 0.55	0.65 ± 0.04
	21	1.30 ± 0.09	0.33 ± 0.02	1.64 ± 0.12	3.94 ± 0.16	0.70 ± 0.06
**T_3_**	0	1.02 ± 0.01	0.32 ± 0.00	1.34 ± 0.03	3.20 ± 0.25	0.51 ± 0.06
	7	1.42 ± 0.15	0.35 ± 0.01	1.78 ± 0.14	4.07 ± 0.63	0.55 ± 0.06
	14	1.51 ± 0.06	0.38 ± 0.01	1.90 ± 0.10	4.10 ± 0.78	0.68 ± 0.04
	21	1.54 ± 0.17	0.38 ± 0.05	1.92 ± 0.16	3.94 ± 0.50	0.69 ± 0.06
**T_4_**	0	1.02 ± 0.01	0.32 ± 0.01	1.34 ± 0.01	3.18 ± 0.05	0.53 ± 0.03
	7	1.45 ± 0.06	0.36 ± 0.04	1.82 ± 0.07	4.02 ± 0.08	0.55 ± 0.01
	14	1.61 ± 0.20	0.39 ± 0.01	2.01 ± 0.15	4.12 ± 0.48	0.70 ± 0.02
	21	1.66 ± 0.17	0.38 ± 0.04	2.05 ± 0.21	4.37 ± 0.34	0.72 ± 0.04
**T_5_**	0	1.02 ± 0.05	0.32 ± 0.01	1.34 ± 0.04	3.19 ± 0.25	0.52 ± 0.01
	7	1.44 ± 0.10	0.35 ± 0.02	1.79 ± 0.09	4.16 ± 0.70	0.53 ± 0.06
	14	1.47 ± 0.07	0.35 ± 0.01	1.82 ± 0.07	4.20 ± 0.34	0.60 ± 0.02
	21	1.45 ± 0.02	0.34 ± 0.01	1.80 ± 0.04	4.26 ± 0.17	0.65 ± 0.01
**ANOVA**	*F*- values					
	D	49.67*	14.12*	47.87*	11.98*	11.41*
	T	27.24*	3.49*	27.28*	5.5*	ns
	D × T	4.83*	ns	4.79*	ns	ns

From the field-grown plants, at regular intervals (D0, D7, D14 and D21) leaves were sampled for analyses. Values are the means of two seasons (± SD). The *F*-values for ANOVA test are reported. *, significance at *P *< 0.05 level; ns, non significant

### Effect of PBZ on morphology, growth and inflorescence

**Figure 2:**
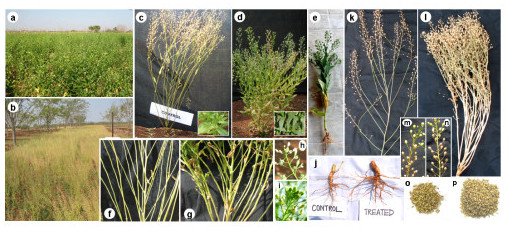
**Comparative morphology of treated and control plants**. Two different phenological stages of field grown *Camelina sativa *cv. Celine in our experimental plot showing (a) onset of fruit setting stage and (b) full silique ripened stage (when the plants were ready to harvest). Comparative plant morphology of (c) T_0 _and (d) T_4 _plants showing reduced height and compact appearance of *Camelina *in T_4 _conditions whereas, the T_0 _plants were taller exhibiting normal growth pattern. The PBZ treated plants (T_4_) showed delayed senescence and possessed dark green leaves (d: inset) when compared to the light green, early senescing leaves of control (c: inset). The highest concentration of PBZ (T_5_) caused severe undesirable changes in growth and morphology of *Camelina *with drastic reduction in yield characteristics (e). PBZ treatment also modified the branching pattern of *Camelina*: (g) T_4 _plants showed increased number of axillary branches and stem thickening when compared to (f) untreated control. Inflorescence in (i) T_4 _plant was a compact raceme with more number of clustered flowers whereas, in (h) untreated T_0 _plants, we observed normal raceme with less number of flowers. (j) PBZ treatment caused significant changes in root growth and morphology exhibiting better rooting vigour (in terms of root biomass and volume) in T_4 _plants when compared to T_0_. Mature T_4 _plants possessed significantly higher number of siliques showing improved silique yield per plant (l, n, p) whereas, the controls (k, m, o) had less of number of siliques and concurrently yielded low.

The PBZ application significantly decreased (p < 0.001) plant height when compared to control and induced dwarfing effect in both trials. Plant height was found to be 10.7%, 18.4%, 24.3% and 35.1% less in T_1_, T_2_, T_3 _and T_4 _plants, respectively when compared to controls (T_0_). The highest concentration of PBZ (T_5_) showed maximum reduction (47.5% decrease) in plant height with respect to control. The influence of PBZ on plant height was represented in Figure 2, which depicts the comparative difference in heights among T_5 _(Figure 2e), T_4 _(Figure 2d) and T_0 _(Figure 2c) plants. In treatments including T_3 _and T_4_, reduction in plant height was accompanied by significant increase (p < 0.001) in branch number and thickening of branches. Figure 2 shows the comparative morphology of branches between control (Figure 2f) and T_4 _(Figure 2g) plants. An increase in 24.3%, 8.9% and 2.4% branch number was recorded in T_4_, T_3 _and T_2 _respectively when compared to control plants (Table 2). However, T_5 _plants showed reduced stem growth (25%) and branch number (Figure 2e) (Table 2). PBZ treatments significantly (0.001) enhanced the accumulation of dry matter in stem when compared to control. At final harvest, the increase in stem dry biomass (excluding the siliques and leaf dry weights) recorded for the treatments T_1_, T_2_, T_3 _and T_4 _were about 8.5%, 23.8%, 39.8% and 57.5%, respectively when compared to controls. Stem dry biomass content between T_5 _and T_0 _plants did not vary significantly.

**Table 2:** Treatment effects of paclobutrazol on morphology and growth characteristics of *Camelina sativa*

	**Traits**							
	
**Treatments**	**Plant** **height** **(cm)**	**Branches** **plant**^**-1**^	**Stem dw** **plant^-1 ^(g)**	**Inflorescence** **type**	**Number of flowers** **raceme**^**-1**^	**Duration of** **leaf 'stay green'** **after fruit setting** **(days)**	**Root length** **(cm)**	**Root dw** **plant^-1 ^(g)**
	
**T_0_**	86.8 ± 0.48^a^	24.6 ± 2.45^a^	11.85 ± 0.08^a^	NR	30.42 ± 2.37^a^	BP (8 ± 2d)	15.7 ± 0.14^a^	1.75 ± 0.07^a^
**T_1_**	78.4 ± 0.92^b^	24.8 ± 2.04^a^	12.86 ± 0.14^b^	NR	31.04 ± 2.52^b^	BP (8 ± 2d)	15.3 ± 0.21^a^	1.80 ± 0.02^a^
**T_2_**	70.8 ± 0.62^c^	25.2 ± 1.61^b^	14.68 ± 0.09^ab^	NR	31.20 ± 2.44^b^	BP (8 ± 2d)	15.8 ± 0.10^a^	2.14 ± 0.04^b^
**T_3_**	65.7 ± 0.55^d^	26.8 ± 1.03^c^	16.57 ± 0.12^c^	CR	33.11 ± 2.30^c^	PP (14 ± 2d)	15.6 ± 0.15^a^	2.48 ± 0.01^ab^
**T_4_**	56.3 ± 0.81^e^	30.6 ± 1.64^d^	18.67 ± 0.08^bc^	CR	42.78 ± 2.48^ab^	PP (15 ± 2d)	15.8 ± 0.20^a^	2.61 ± 0.02^c^
**T_5_**	45.6 ± 0.42^f^	18.8 ± 1.22^e^	11.45 ± 0.04^a^	CR	20.05 ± 1.08^d^	PP (18 ± 2d)	14.2 ± 0.20^b^	1.62 ± 0.07^bc^

Field-grown plants were observed regularly for any change in morphological traits (inflorescence type, duration of leaf 'stay-green') during the study period and measurements were taken on selected growth parameters (Plant height, branches per plant, stems dry weight, number of flowers per raceme, root length and root dry weight per plant). Values are the means of two seasons (± SD). Different letters indicate that the values are significantly different at p < 0.001. NR: normal raceme; CR: compact raceme; BP: brief period; PP: prolonged period

The most striking effect of PBZ was compaction of inflorescence. Compared to T_0_, T_1 _and T_2 _plants which exhibited normal racemes, higher concentrations of PBZ (T_3_, T_4 _and T_5_) induced conspicuous changes in inflorescence morphology which resulted in compact raceme with less distance between two floral nodes when compared to normal raceme. Figure 2 represents the comparative differences in raceme morphology between T_0 _(Figure 2h) and T_4 _(Figure 2i) plants.

The number of flowers per raceme was significantly increased (p < 0.001) in PBZ treated plants (except T_5 _which showed apparent reduction in number of flowers per raceme) more specifically in case of T_3 _and T_4_. The relative increase in number of flowers per raceme was 40.6% in T_4_, followed by T_3 _(8.8%) and T_2 _(2.6%) in respect to control (Table 2).

The leaves of PBZ treated plants were dark green (Figure 2d, inset) and the leaf 'stay-green' persisted for a prolonged time period even after fruit setting in both trials (Table 2). Plants treated with higher PBZ concentrations (T_3_, T_4 _and T_5_) also had a longer leaf life span when compared to control. In contrast to PBZ treated plants, the T_0 _plants had yellowish-green leaves (Figure 2c, inset) which were in 'stay-green' state for a brief period of time after fruit setting. Moreover, the leaf life span of T_0 _plants was less and showed senescence followed by defoliation with progress in fruit ripening. Figure 2 clearly elucidates the comparative difference in leaf 'stay-green' phenomenon between the control (Figure 2c) and T_4 _(Figure 2d) plants during the same time of growth. The figure also depicts that during fruit ripening stage, the T_4 _plants still had substantial green foliage, whereas in case of T_0_, only a few senescing leaves were left in the branches and most of the foliage was already lost.

The PBZ application also resulted significant (p < 0.001) increase in root dry weight in T_2 _(22.8%), T_3 _(41.7%) and T_4 _(49.2%) plants. However, further increase in PBZ concentration (T_5_) showed significant (p < 0.001) reduction (7.5%) in root dry weight when compared to control (T_0_) plants (Table 2). No significant changes in root length were observed among the treatments except in case of T_5 _where a decrease of ~9.5% in root length was recorded compared to control (Table 2). Figure 2j clearly depicts the comparative root morphology of control (T_0_) and treated (T_4_) plants.

### Effect of PBZ on fruiting and seed yield

PBZ application induced significant changes in fruiting and various yield related characteristics of *Camelina *over the control in both trials (Table 3). The number of siliques borne by a single plant differed significantly (p < 0.001) among the treatments. The control plants possessed ~733.8 siliques per plant, while with the increase in concentration of PBZ (T_1_, T_2_, T_3 _and T_4_), we observed considerably higher number of siliques per plant. The highest number of siliques were obtained in T_4 _plants (1285.6) followed by T_3_, T_2 _and T_1_. Figure 2 shows matured control plants (Figure 2k) and T_4 _(Figure 2l) with ripened siliques adhered to branches elucidating silique density in T_4 _(Figure 2m, n). PBZ treatments did not cause any increment in the number of seeds per silique, rather the seed number per silique was found to decrease with increase in the concentration of PBZ. There was no appreciable change in 100 silique weight among the treatments T_0_, T_1 _and T_2_; however, T_3 _and T_4 _plants showed a significant (p < 0.001) decrease of about 25% and 28% in 100 silique weight, respectively. Most severe reduction in silique weight was detected in T_5 _plants exhibiting a reduction of 51.7% in 100 silique weight when compared to control. PBZ treatments did not show any appreciable change in 100 seeds weight among the treatments T_0_, T_1 _and T_2_. However, a reduction (p < 0.001) of 7.4%, 9.6% and 11.7% in weight of 100 seeds were determined in case of T_3_, T_4 _and T_5 _plants, respectively. The data on Shell weight (SHW):Seed weight (SW) indicated significant (p < 0.001) difference among the treatments. With the increase in concentration of PBZ, we found considerable reduction (p < 0.001) in SHW:SW. The highest SHW:SW was noted in T_0 _followed by T_1_, T_2_, T_3_, T_4 _and T_5 _(Table 3). Seed yield per plant increased significantly (p < 0.001) in T_1_, T_2_, T_3 _and T_4 _plant when compared to T_0 _(Table 3). Figure 2 shows comparable seed yield between control (Figure 2o) and T_4 _(Figure 2p) plants exhibiting substantially high seed yield per plant in T_4 _than compared to T_0_. Further increment in PBZ concentration (T_5_) strongly decreased seed yield per plant with a reduction of 34.5% in respect to control. The relative increase (p < 0.001) in seed yield (g m^-2^) obtained with T_1_, T_2_, T_3 _and T_4 _were 3.91%, 8.53%, 16.58% and 74.23%, respectively when compared to control. In case of T_5_, a substantial (p < 0.001) reduction of 38.1% was obtained in seed yield (g m^-2^) when compared to control plants.

**Table 3:** Treatment effects of paclobutrazol on yield traits of *Camelina sativa*

	Yield traits						
	
Treatments	**No. siliques plant**^**-1**^	**No. seeds silique**^**-1**^	Siliques weight (100 fruits^-1^) (g)	Seed weight (100 seeds^-1^) (g)	SHW:SW	Seed yield plant^-1 ^(g)	Seed yield (g m^-2^)
**T_0_**	733.8 ± 27.12^a^	19.2 ± 1.47^a^	2.98 ± 0.12^a^	0.094 ± 0.01^a^	0.652 ± 0.01^a^	7.44 ± 0.16^a^	130.65 ± 11.78^a^
**T_1_**	755.6 ± 21.60^b^	17.8 ± 1.31^b^	2.76 ± 0.23^a^	0.094 ± 0.02^a^	0.637 ± 0.06^b^	8.12 ± 0.15^b^	135.76 ± 6.39^b^
**T_2_**	770.6 ± 41.17^c^	16.4 ± 0.84^c^	2.66 ± 0.39^a^	0.093 ± 0.02^a^	0.614 ± 0.08^c^	8.32 ± 0.24^b^	141.80 ± 11.83^c^
**T_3_**	873.6 ± 64.60^d^	14.6 ± 0.51^d^	2.23 ± 0.18^b^	0.087 ± 0.03^b^	0.582 ± 0.07^d^	9.08 ± 0.34^c^	152.32 ± 11.12^d^
**T_4_**	1285.6 ± 20.33^e^	13.4 ± 1.50^e^	2.12 ± 0.16^c^	0.085 ± 0.02^c^	0.508 ± 0.03^e^	13.54 ± 0.27^d^	227.64 ± 68.69^e^
**T_5_**	574.8 ± 35.72^f^	10.8 ± 1.22^f^	1.44 ± 0.18^d^	0.083 ± 0.01^d^	0.333 ± 0.02^f^	4.87 ± 0.43^e^	80.84 ± 11.90^f^

Treatment effects of paclobutrazol on seed yield of *Camelina sativa*. Field-grown plants were harvested completely at the end of the study. Harvested ripened siliques were carefully preserved in plastic packets, carried to laboratory and selected yield parameters (siliques per plant, seeds per silique, silique weight, seed weight, SHW:SW, seed yield per plant and seed yield per unit area) were measured. Values are the means of two seasons (± SD). Different letters indicate that the values are significantly different at p < 0.001. SHW: shell weight; SW: seed weight

### Effect of PBZ on oil yield and protein content

**Figure 3:**
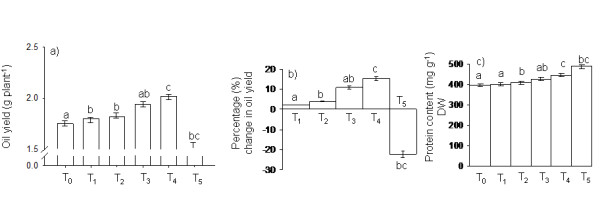
**Oil yield and protein content in *Camelina *after treatment with Paclobutrazol**. Effect of increasing concentrations of PBZ treatment on (a) oil yield per plant and (b) percentage change in oil yield with respect to control (c) protein content in seeds (expressed on a dry weight basis). Values are mean of two trials ± SD. Different letters indicate that the values are significantly different at p < 0.05 level.

Application of PBZ resulted in significant changes in the oil yield of *Camelina *in both trials (Figure 3a). In case of control plants (T_0_), the oil yield was found to be 1.75 g plant^-1 ^and the T_1 _plants also did not differ significantly in oil yield plant^-1 ^when compared to control. However, with further increase in the concentration of PBZ (T_2_, T_3 _and T_4_), an increasing trend in oil yield was monitored and the oil yield was recorded to be 1.82 g, 1.94 g and 2.02 g plant^-1 ^in T_2_, T_3 _and T_4 _plants, respectively. The T_4 _plants showed the maximum increment of 15.41% (p < 0.05) in oil yield when compared to controls (Figure 3b). The highest PBZ concentration (T_5_) significantly decreased (p < 0.05) oil yield of *Camelina *to a large extent, which resulted to nearly 22.2% decrease in oil yield plant^-1 ^when compared to the yield in control plants (T_0_) (Figure 3b). Application of PBZ resulted in increase in protein content in the seeds in both the trials (Figure 3c). In case of control plants (T_0_), the protein content was found to be 396.82 mg g^-1^. In T_1_, T_2 _and T_3 _plants, the protein content did not differ significantly when compared to controls and were recorded to be 400.44 mg, 408.26 mg, and 427.38 mg g^-1 ^respectively. However, with further increase in the concentration of PBZ (T_4 _and T_5_), a significant increase (p < 0.05) in protein content was observed and it was found to be 445.74 mg and 486.74 mg g^-1 ^respectively.

## Discussion

Synthetic growth retardants, when applied in appropriate concentrations have shown tremendous influence on plant growth, development and productivity. PBZ induced changes in growth and productivity of agronomical and horticultural crops had been described in some of the recent studies [7-10]. These reports indicated the potential of PBZ in controlling vegetative growth, vigour and consequent advantages in increasing flowering, yield and fruit quality in different crop plants including horticultural crops. In the present study, we document the morpho- physiological, growth and yield responses of *Camelina *after PBZ treatment. We also provide a positive connotation towards the use of PBZ for yield improvement in *Camelina*.

Seed yield in *Camelina *relies on a number of yield attributing traits including number of flowering branches, number of siliques plant^-1^, number of seeds silique^-1^, siliques weight, 100 seed weight and seed yield plant^-1^. In the present study, except T_5 _(125 mg l^-1^), other concentrations (particularly T_3_: 75 mg l^-1 ^and T_4_: 100 mg l^-1^) showed quantitative enhancement in most of the yield attributing traits which contributed towards overall seed and oil yield improvement in *Camelina*. Severe and undesirable loss in seed and oil yield was observed when the plants were treated with higher PBZ concentration (T_5_: 125 mg l^-1^). Reduced yields were recorded in peanut [11] and *Jatropha *[10] associated with higher PBZ concentrations. Our data suggest that optimizing PBZ dose is a prerequisite for any such yield improvement programmes and implies that PBZ dose between 75 mg l^-1 ^to 100 mg l^-1 ^can effectively improve the economic traits, including higher seed and oil yields in *Camelina*. In our study, PBZ was applied only once, (at the onset of inflorescence) which enhanced mainly the number of siliques per plant rather than seed number per silique and seed weight. More siliques and increased seed yields were also reported in *Brassica *after PBZ treatment [12], although not to the extent we report here in *Camelina*. They applied PBZ twice: once at the green floral bud stage of the terminal raceme and then after one week interval. Berova and Zlatev [8] also showed increment in tomato yield due to PBZ treatment and illustrated that soil application showed better yields compared to foliar application. However, the yield increment was only 26.9% higher and not as high as in case of *Camelina *in our experiment. Hence, the time of application, dosage and phenological stage of plant and type of PBZ treatment (foliar/soil) appear to be critical for yield increment in crops like *Camelina *[11].

**Figure 4:**
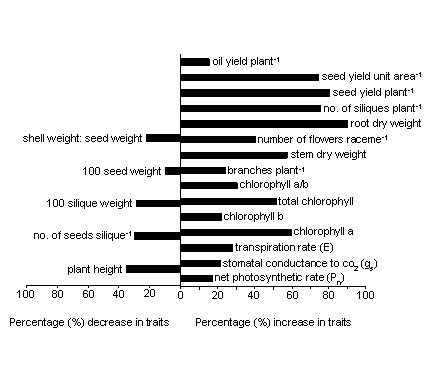
**Comparative changes in growth, physiological and yield traits of *Camelina sativa *due to PBZ treatment (T_4_) with respect to untreated control (T_0_)**. Bars facing right and left sides of the plot indicate percentage (%) increase and decrease in traits, respectively.

Several morphological, growth and biochemical attributes are reported to be linked with plant productivity, yield and harvest index (HI). In the present investigation, the results suggest that the effect of PBZ on seed yield improvement (including oil yield) might be linked to improved CO_2 _assimilation physiology, enhanced sink activity, better partitioning coefficient, quantitative enhancement of yield determining growth traits, altered phenology, better plant canopy and rooting vigour (Figure 4). The effects of PBZ on leaf assimilation physiology has received less attention and the existing information show varied responses in leaf gas exchange traits after PBZ treatments [8,11,13]. We observed a positive correlation between photosynthetic rates and yield of *Camelina *suggesting a possible involvement of carbon assimilation in enhancing the yields in PBZ - treated plants [13]. We noticed a rapid response and significantly higher enhancement in photosynthetic traits within a week after PBZ application. The positive effect of PBZ on photosynthetic processes may not be single, rather conjugated effect of multiple factors. Gao et al. [14] found that the PBZ treatment increased chloroplast size in plants. PBZ - induced changes in the photosynthetic activity of chloroplasts was also reported by Christov et al. [15]. Such positive changes in chloroplast structure and function can dramatically improve CO_2 _fixation process in plants and might be involved in enhancing the photosynthetic ability of PBZ treated *Camelina *plants, as observed in our present study. Kende [16] reported enhanced cytokinin biosynthesis in roots after PBZ application. In our study, we found *g_s _*to be positively correlated to *P_n _*which implies better stomatal functioning in PBZ treated plants when compared to controls [17].

Increased contents of photosynthetic pigments have been considered to be a precondition for higher photosynthesis. In our study, the foliage of PBZ treated plants displayed intense dark green color which was positively correlated to increased chlorophyll contents. Dalziel and Lawrence [18] reported increment in cell volume in sugar beet, treated with PBZ without any increase in the number of chloroplasts per cell but an increase in chlorophyll per unit leaf area indicating that PBZ increased the chlorophyll content per chloroplast. Similar reports on different crops depicted that the foliage of PBZ-treated plants displayed an excessive dark green color due to enhanced chlorophyll synthesis [8,19-21]. Earlier reports indicated that the increased chlorophyll content was actually enhanced due to phytyl, an essential part of the chlorophyll molecule, which is generated via the same terpenoid pathway as gibberellins. PBZ is known to block the production of gibberellins, resulting in utilization of intermediates of gibberellin synthesis to facilitate increased phytyl production [22] which should have resulted in higher chlorophyll content and dark green leaf color as was observed in our PBZ treated *Camelina *plants.

An important phenological change that was observed in our study was that PBZ delayed senescence and extended period of 'stay-green' in *Camelina*. An increase in the levels of cytokinins and polyamines over the senescence promoting hormones ABA and ethylene has been reported in plants treated with growth retardants [23-26]. We believe that PBZ has delayed the onset of senescence in *Camelina *leaves probably by enhancing the endogenous levels of cytokinins promoting chlorophyll formation, increased activity of certain antioxidant enzymes [27] and retard senescence. A longer 'stay-green' condition might have concurrently enhanced the duration of leaf photosynthesis in PBZ treated plants by keeping the leaves photosynthetically active for a longer time which in turn might have contributed to better plant productivity in *Camelina*. In our study, the PBZ treated plants senesced slowly compared to the control plants. As cytokinins are known to retard senescence by preventing protein degradation and promoting protein synthesis which should have resulted an increase in seed protein content as observed in this present investigation [28].

In our experiment, PBZ caused significant changes in the "morphological components" of growth and productivity. PBZ treatment resulted in reduced gibberellin (GA) biosynthesis and shortened the height of plants [6,8,12,29]. In our study, PBZ treatments were effective in reducing plant height of *Camelina *and also modified the branching patterns in terms of their increased number and stem thickening. However, severe retardation of growth was reflected in plant height, branch and canopy size in the current study when the plants were sprayed with higher PBZ concentration (T_5_: 125 mg l^-1^) which was also reported earlier [10,30,31]. PBZ treatments modified the branching pattern in *Camelina *as it showed a compact appearance with reduced internodal distance. Increased branching, as observed in the PBZ treated *Camelina *plants (T_3 _and T_4_), might be due to greater metabolic and divisional activity in the shoot apical meristem regions. Increased branching also implies that PBZ caused apparent changes in apical dominance of *Camelina *and concomitantly induced lateral bud initiation. Thus, PBZ induced suppression of GA biosynthesis should have resulted in changes in hormonal constellation favourable for axillary bud initiation and branching as observed in our present study [32]. The change in canopy coverage due to broader canopy development might have facilitated improved light interception for better photosynthesis in leaves and stems of PBZ treated *Camelina *plants. Further, as mentioned before, the leaves in PBZ treated plants were closely packed, dark green and remained on plants for a larger period than controls. This may explain increased dry matter accumulation in stem and root and simultaneous yield increments despite reduced plant height due to PBZ treatments. An improved rooting vigour as observed in T_3 _and T_4 _plants might have facilitated better hydraulic conductance and leaf water status which can be also linked to better *P_n _*and associated gas exchange functions and dry matter accumulation in PBZ treated *Camelina *plants.

In this study, the variation in the intensity of flowering response between treated and control *Camelina *plants has provided a strong evidence in favour of PBZ application for maximizing the potential for flowering of *Camelina*. Our results demonstrate more number of flowers per plant with increasing concentration of PBZ (between 50 - 100 mg l^-1^) when compared to controls. External application of plant growth regulators also play a role in floral sex determination [33]. There are reports that indicate that PBZ can noticeably enhance the total phenolic content of terminal buds, alter the phloem to xylem ratio of the stem and increase total non structural carbohydrates (TNC) [34,35]. These adjustments could be essential in promoting flowering by amending assimilates partitioning and patterns of nutrient supply for new growth. Increased availability of assimilates can possibly lead to initiation of more floral buds on the branches which ultimately contribute to greater seed yield [12]. The proportion of photoassimilates allocated towards reproductive organs could have a direct effect on flowering, fruit and seed yield. Plant growth regulators are known to promote distribution of photoassimilates vis-à-vis meristematic activity. Addo-Quaye et al. [36] elucidated that PBZ changed the pattern of assimilate distribution towards reproductive parts, especially to the terminal and upper branches of plants thus, increasing their sink capacity leading to higher silique formation. In our present study too, the increased availability of assimilates due to increased *P_n _*might have resulted in more siliques on branches and ultimately resulted in higher seed yield in PBZ treated *Camelina *plants. Since there was more number of seeds per plant in the PBZ treated *Camelina *plants (excluding T_5_), we concomitantly recorded a simultaneous improvement in oil yields.

## Conclusions

On the basis of our field based micro-trial experiments, the following conclusions can be ascertained: reduced plant height, more number of thick axillary branches and improved rooting vigour are significant phenological characteristics after PBZ treatment in *Camelina*; PBZ improved CO_2 _assimilation physiology, enhanced total chlorophyll content and retarded senescence causing higher biomass accumulation and silique yield in *Camelina*; the total number of siliques and seed yield per plant were significantly high in PBZ treated plants, which in turn caused increased oil yields. Our findings depict that PBZ treatment can be largely effective in improving the seed and oil yield of this promising biofuel energy crop and has potential application to improve *Camelina *yield, even under resource limited agroclimatic conditions.

## Methods

### Plant material, study site and experimental design

High quality, disease free seeds of *Camelina sativa *(cv. Celine) were used in this study. A field micro-trial set up was established in the experimental farm of Tree Oils of India Limited (TOIL), Zaheerabad, Medak district, Andhra Pradesh (latitude 17°36' N; longitude 77°31' E; 622 m MSL) during winter season. This study was performed twice for a duration of five months (October 2010 to February 2011). The site has a tropical, a hot steppe agroclimate. However, during winter season (October to February), the climatic conditions are cool and moderate. Climatic data were collected from a weather station adjacent to the experimental field. Soil samples were collected randomly from the experimental plot in each month and were analyzed for physicochemical properties. Meteorological features and important soil characteristics were recorded at the experimental site during our experiment and were presented in Additional file 1: Table S1.

**Figure 5:**
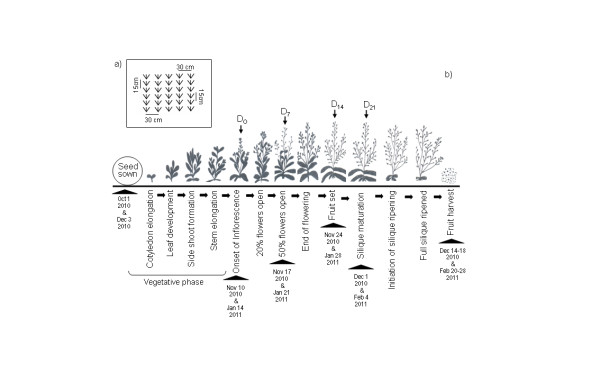
**Diagram elucidating experimental design**. Diagrammatic representation of (a) experimental plot with inter-row and inter-plant spacing, showing (b) phenological growth stages of *Camelina sativa*. The diagram illustrates the time course of the experiment which includes date of seed sowing, vegetative and reproductive growth phases, different sampling dates and time of harvest. PBZ treatments were applied on January 14, 2011 (Day 0: D_0_) with the initiation of flowering. Before PBZ application, the first leaf sampling and measurements were conducted. Thereafter, on Day 7, 14 and 21 (D_7_, D_14_, and D_21 _respectively), plants were measured and sampled for various growth, physiological and biochemical characteristics. Yield harvesting was conducted in the last week of February 20-28, 2011

The experiment was conducted in a randomized block design with three replications. Land was prepared with ploughing followed by proper levelling. Each plot had 30 cm inter-row spacing and 15 cm distance between the plants within each row (Figure 5). The seeds were sown on October 1, 2010; the vegetative growth phase continued till 2^nd ^week of November, 2010 followed by the onset of inflorescence. On November 10, 2010 (day 0: D_0_), with the onset of flowering, paclobutrazol (PBZ) treatments were applied. Before application, on D_0_, the leaves were measured and sampled for various physiological and biochemical characteristics. Thereafter, on day 7, 14 and 21 of treatment (D_7_, D_14 _and D_21_, respectively) at regular intervals of seven days, the control and treated plants were measured and sampled for various growth, physiological and biochemical characteristics. Finally, fruit harvest was conducted from December 14 to December 18, 2010 when the siliques of all the treatments were found to be completely ripened (Figure 5). The second trial was done with the seed sown on December 3, 2010; the vegetative growth phase continued till 2^nd ^week of January, 2011 followed by the onset of inflorescence. On January 14, 2011 (day 0: D_0_), with the onset of flowering, PBZ treatments were applied. Finally, fruit harvest was conducted from February 20 to February 28, 2011 when the siliques of all the treatments were found to be completely ripened (Figure 5).

### Treatment application and agronomical practices

The commercial product CULTAR [active ingredient paclobutrazol 25.0% (w/v) Syngenta Ltd., UK] was used. Five different soil treatments were applied: 0 (control: T_0_); PBZ: 25 mg l^-1 ^(T_1_); PBZ: 50 mg l^-1 ^(T_2_); PBZ: 75 mg l^-1 ^(T_3_); PBZ: 100 mg l^-1 ^(T_4_) and PBZ: 125 mg l^-1 ^(T_5_). PBZ was applied at the base of the stem at the rate of 50 ml solution plant^-1^. The control (T_0_) plants received the same amount of distilled water. All experimental plots were fertilized with a basal application of N, P and K at the rate of 100 kg urea, 30 kg single super-phosphate (SSP) and 30 kg muriate of potash (MOP) per hectare. Half of the urea and whole amount of other fertilizers were applied at final land preparation stage. Remaining half of urea was applied after 15 days of seed sowing. Surface irrigation of approximately 30 mm was applied at regular intervals to retain soil moisture content of 80-85%. Mechanical weeding was done once in a week and other intercultural operations including hoeing, tilling, earthing up etc were also undertaken at regular intervals. All culture practices were the same in all plots.

### Leaf gas exchange measurements

Fully expanded, healthy leaves at the 3^rd ^and 4^th ^position from the top were used for photosynthetic leaf gas exchange analyses. Measurements were confined to the period between beginning of canopy closure and onset of crop senescence. Ten representative plants under each treatment were tagged and were repeatedly used on D_0_, D_7_, D_14 _and D_21 _for gas exchange measurements during 09.00 to 11.00 solar hour using a portable infra-red gas analyser (*IRGA*) (LCpro-32070, ADC Bioscientific, UK) at a saturating PAR of 1500 μmol m^-2 ^s^-1^. Net photosynthetic rate (*P_n_*), stomatal conductance to CO_2 _(*g_s_*) and transpiration rate (*E*) were measured. While measuring, the air humidity inside the leaf chamber was about 60%, CO_2 _concentration was 370 μmol mol^-1 ^and air temperature was 25 ± 2°C. Each measurement was made when the readings of net photosynthetic rate (*P_n_*) were stabilized; this process typically took 1 to 2 min.

### Measurements of chlorophyll and carotenoid contents

Chlorophyll and carotenoid contents were estimated following the methodology of Hiscox and Isrealstam [37]. Fresh leaf samples (25 mg) were homogenized in 10 ml of di-methyl sulphoxide (DMSO) and were kept in hot water bath at 60°C for 1 h for complete pigment extraction. The extracts were then cooled in dark and OD values were recorded at 663 nm, 645 nm, 480 nm and 510 nm using a UV-Visible spectrophotometer (Shimadzu 1800, Japan). The amount of chlorophyll a, chlorophyll b, total chlorophyll and total carotenoids were calculated according to Arnon [38].

### Plant morphology: Vegetative growth, inflorescence and fruit related development

After PBZ application, at a regular interval of 7 days, the control and treated plants were photographed to record and compare the morphological differences among the plants due to different doses of PBZ treatment. Plant height was regularly evaluated (weekly once) by considering 30 randomly distributed plants per treatment. The onset of flowering was registered for all treatments. The inflorescence intensity, raceme type, size of raceme and numbers of flowers per raceme were observed with the number of days passed after treatment application. For all treatments, the duration of leaf 'stay-green', number of branches per plant and the progressive growth of siliques were carefully monitored from the onset of flower opening until maturity (when the siliques became ripened). At the end of the experimental period, 10 randomly distributed plants were up-rooted, the soil was gently washed from roots, roots were photographed and the morphological differences (related to root growth pattern) were recorded. Stem and root fresh weights were measured and thereafter dried in an incubator at 70°C for 4 - 5 days till constant weight was recorded.

### Measurement of seed yield characteristics and oil yield

When the siliques were mature, the plants were harvested (during both trials). The seeds were sun dried and thereafter data on various seed yield parameters including number of siliques per plant, number of seeds per silique, 100 silique weight, 100 seed weight, shell weight: seed weight (SHW:SW), total seed yield per plant and area based seed yield were recorded.

Oil was extracted from seeds by Soxhlet method using hexane as a solvent (distillation temperature range = 55-70°C) according to AOAC [39]. Seeds were ground in a coffee grinder to make powder. Oil was extracted with 150 ml of hexane at distillation temperature for 2 to 3 h in the Soxhlet extractor using a heating mantle. Hexane was removed from the extracted oil using a rotary evaporator (Heidolph 514-01002-06-0, Germany) at 55°C under reduced pressure for 30 min.

### Measurement of protein content in seeds

Total protein was extracted [40] and estimated [41] from harvested seeds dried in an incubator at 40°C for 4-5 days. Protein content was reported on a dry weight basis.

### Statistical analysis

Results were represented as mean of two trials ± SD (standard deviation) of n (n = 6-15) measurements. Data were analysed using one way and two way analysis of variance (ANOVA) to test significant differences within days (D), among the PBZ treatments (T) as well as for their interactions (D × T) on measured traits. Whenever significant differences were detected, means were separated using least significant difference (LSD) test at the 5% level of significance. All statistical analyses were performed using the program SIGMA PLOT 11.0.

## Competing interests

The authors declare that they have no competing interests.

## Authors' contributions

ARR conceived and designed the investigations, coordinated the experiments and drafted the manuscript. SK, BSKC and JS performed the experiments on the field and analysed the data. AG was involved in statistical analysis of the data and preparing graphs and figures. SG participated in preparing the field for plantation and maintenance of plant growth. All authors read and approved the final manuscript.

## Supplementary Material

Additional file 1 **Table S1 Climatic conditions and selected soil characteristics of the study site during experimental time span (Oct 2010-Feb 2011)**. Click here for file
