# Supplementary material for: Paclobutrazol treatment as a potential strategy for higher seed and oil yield in field-grown camelina sativa L. Crantz
Source: BMC Res Notes. 2012 Mar 13;5:137. doi: 10.1186/1756-0500-5-137 (PMC3320555; doi:10.1186/1756-0500-5-137)
Supplement: Additional file 1 — Table S1 Climatic conditions and selected soil characteristics of the study site during experimental time span (Oct 2010-Feb 2011). [file 1756-0500-5-137-S1.DOC]

**Supplementary Table 1 - Climatic conditions and selected soil characteristics of the study site during experimental time span (Oct 2010-Feb 2011)**

|  | **Experimental months** | | | | |
| --- | --- | --- | --- | --- | --- |
|  | **October 2010** | **November 2010** | **December 2010** | **January 2011** | **February 2011** |
| **Climatic conditions** |  |  |  |  |  |
| **Mean max. temperature (ºC)** | 27.2 | 25.8 | 28.4 | 30.2 | 31.9 |
| **Mean min. temperature (ºC)** | 22.9 | 17.4 | 14.6 | 13.1 | 16.9 |
| **Relative humidity (%)** | 72 | 74 | 56 | 42 | 43 |
| **Mean rainfall (cm)** | 0.04 | 0.01 | 0.0 | 0.0 | 0.01 |
| **Atmospheric CO_2_ concentration (μmol m^-2^ s^-1^)** | 378.4 | 378.2 | 378.6 | 377.5 | 378.2 |
| **Wind velocity (km h^-1^)** | 5 | 5 | 4 | 4 | 5 |
| **Mean photosynthetic flux (µmol m^-2^ s^-1^)** | 1004 | 1005 | 1007 | 1004 | 1007 |
| **Soil characteristics (at 0–30 cm soil depth)** |  |  |  |  |  |
| **Texture** | Sandy loam | Sandy loam | Sandy loam | Sandy loam | Sandy loam |
| **pH** | 7.2 | 7.2 | 7.2 | 7.2 | 7.2 |
| **Organic carbon (%)** | 0.7 | 0.7 | 0.7 | 0.7 | 0.7 |
| **Available N (kg ha^-1^)** | 120 | 120 | 120 | 120 | 120 |
| **Available P (kg ha^-1^)** | 1.2 | 1.2 | 1.2 | 1.2 | 1.2 |
| **Available K(kg ha^-1^)** | 115.4 | 115.4 | 115.4 | 115.4 | 115.4 |
